# Circular RNA MELK Promotes Chondrocyte Apoptosis and Inhibits Autophagy in Osteoarthritis by Regulating MYD88/NF-*κ*B Signaling Axis through MicroRNA-497-5p

**DOI:** 10.1155/2022/7614497

**Published:** 2022-07-30

**Authors:** Yingchi Zhang, Rui Lu, Xiaojian Huang, Enzhi Yin, Yong Yang, Chengla Yi, Hongbo You, Xianzhou Song, Xuefeng Yuan

**Affiliations:** ^1^Department of Traumatology, Tongji Hospital, Tongji Medical College, Huazhong University of Science and Technology, Wuhan, Hubei 430030, China; ^2^Department of Orthopedics, Tongji Hospital, Tongji Medical College, Huazhong University of Science and Technology, Wuhan, Hubei 430030, China

## Abstract

Osteoarthritis (OA) is a rheumatic disease and its pathogenesis involves the dysregulation of noncoding RNAs. Therefore, the regulatory mechanism of circular RNA MELK (circMELK) was specified in this work. OA human cartilage tissue was collected, and circMELK, miR-497-5p, and myeloid differentiation factor 88 (MYD88) expression were examined. Human chondrocytes were stimulated with interleukin- (IL-) 1*β* and interfered with vectors altering circMELK, miR-497-5p, and MyD88 expression to observe their effects on cell viability, cell cycle and apoptosis, autophagy, and inflammation. The binding relationship between RNAs was verified. The data presented that OA cartilage tissues presented raised circMELK and MYD88 and inhibited miR-497-5p expression. IL-1*β* suppressed cell viability, prevented cell cycle, and induced apoptosis, autophagy, and inflammation of chondrocytes. Functionally, IL-1*β*-induced changes of chondrocytes could be attenuated by suppressing circMELK or overexpressing miR-497-5p. circMELK acted as a sponge of miR-497-5p while miR-497-5p was a regulator of MYD88. MYD88 restricted the effect of overexpressing miR-497-5p on IL-1*β*-stimulated chondrocytes. MYD88 triggered nuclear factor-kappaB (NF-*κ*B) pathway activation. Shortly, CircMELK promotes chondrocyte apoptosis and inhibits autophagy in OA by regulating MYD88/NF-*κ*B signaling axis through miR-497-5p. Our study proposes a new molecular mechanism for the development of OA.

## 1. Introduction

Osteoarthritis (OA) is a chronic degenerative joint disease and a major cause of pain and disability, characterized by chronic inflammation, progressive destruction of articular cartilage, and subchondral bone sclerosis [1]. The pathogenesis of OA involves mechanical, inflammatory, and metabolic factors that ultimately lead to the structural destruction and failure of synovial joints [2]. Chondrocyte apoptosis and autophagy are the most important pathological changes in OA [3]. Therefore, exploring the regulatory mechanisms of chondrocyte apoptosis and autophagy may help to develop new strategies for OA treatment.

Circular RNAs (circRNAs), specifically expressed in tissues and cells [4], are an important role in the progression of OA, such as circCDR1as [5], circPDE4D [6], and circRNA_Atp9b [7]. circRNAs exert their biological functions as ceRNAs of microRNAs (miRNAs) to regulate their downstream molecules [8]. A microarray analysis report reveals that hsa_circ_0009127 (circMELK) is one of the top 10 most upregulated circRNAs in postmenopausal osteoporosis patients, which may be involved in autophagy-related pathways, phosphatidylinositol 3-kinase-Akt signaling, forkhead box O signaling, and Ras-mitogen-activated protein kinase signaling [9].

Dysregulation of miRNAs is a key cause of a variety of human diseases, including OA [10, 11], and is a critical role in chondrocyte development and cartilage homeostasis; miRNAs have also been shown to play a role in chondrocyte phenotype through regulating apoptosis, autophagy, and senescence, and regulation of miRNAs in joints is of utility to attenuate OA in animal models [12, 13]. Based on these properties, miRNAs have been proposed as circulating biomarkers for OA. It has been confirmed that miR-497-5p expression is elevated in the serum of OA patients [14]. However, the role of miR-497-5p in OA remains controversial, because another study has described that miR-497-5p expression is decreased in OA cartilage and can attenuate IL-1*β*-induced degradation of chondrocyte cartilage matrix through Wnt/*β*-catenin signaling pathway [15].

We mainly explored the mechanism of circMELK in OA, as well as its interaction with miR-497-5p. Our cell experiments showed that circMELK promotes the apoptosis of OA chondrocytes and inhibits autophagy by regulating the myeloid differentiation factor 88 (MYD88)/nuclear factor-kappaB (NF-*κ*B) signaling axis through miR-497-5p.

## 2. Materials and Methods

### 2.1. Cartilage Tissue Sample

OA human cartilage tissue was isolated from 36 patients who underwent total knee arthroplasty in Tongji Hospital, Tongji Medical College, Huazhong University of Science and Technology. Diagnosis of patients with OA was based on American College of Rheumatology guidelines. The normal human cartilage tissue of femoral condyle and tibial plateau of 36 donors with emergency trauma amputation in Tongji Hospital, Tongji Medical College, Huazhong University of Science and Technology, were used as control, and there was no history of OA or rheumatoid arthritis in those donors. All cartilage tissues were immediately stored at −80°C. All individuals participating were informed of the study and signed informed consent. The protocol of this work was approved by the Ethics Committee of Tongji Hospital, Tongji Medical College, Huazhong University of Science and Technology [16].

### 2.2. Cell Culture and Construction of OA Cell Models

Cartilage specimens were digested with trypsin (Invitrogen, CA, USA) and collagenase type II (Millipore, MA, USA) in Dulbecco's modified Eagle's medium (DMEM) to isolate chondrocytes. Then, the chondrocytes were grown in DMEM/F12 medium containing 10% fetal bovine serum (Thermo, MA, USA), 100 U/ml penicillin, and 100 *μ*g/ml streptomycin (Thermo) and subcultured to passage 3.

Chondrocytes at passage 3 were cultured in DMEM and treated with 10 ng/ml interleukin (IL)-1*β* (Sigma, MO, USA) for 72 h. Cytoplasmic retraction and visible vacuoles are considered the indicators of successful OA modeling [17].

### 2.3. Cell Transfection

circMELK or MYD88 overexpression vector was constructed by Genepharma (Shanghai, China). The circMELK or MYD88 sequence was inserted into the pcDNA3.1 His c plasmid (Genepharma). circMELK or MYD88 without downstream reverse sequence was as the vector group. shRNAs targeting circMELK were constructed into the pGPU6/GFP/Neo vector (Genepharma) to generate sh-circMELK. miR-497-5p mimic, miR-NC, anti-miRNA oligonucleotide (AMO)-497-5p, and AMO-NC were purchased from Genepharma while MYD88 small interfering RNA (si-MYD88) and scramble siRNA (si-NC) were from RiboBio (Guangzhou, China). Chondrocytes at 70% confluence were transfected using Lipofectamine 3000 reagent (Invitrogen). The transfection efficiency was detected 24 h after transfection and all functional experiments were performed 48 h after transfection [18].

### 2.4. Cell Viability Assay

After IL-1*β* stimulation, cell counting kit-8 solution (10 *μ*l/well) was added to the cell culture medium and the optical density_450 nm_ was recorded after 1 h.

### 2.5. Flow Cytometry

Chondrocytes were fixed with ice-cold 70% ethanol and resuspended in a binding buffer to a density of 1 × 10^6^/mL. For cell cycle analysis, cell suspensions were incubated with propidium iodide (PI) (BD Biosciences, NJ, USA). For apoptosis analysis, 10 *μ*L of annexin *V*-fluorescein isothiocyanate and 5 *μ*L of PI were added. A FACSCalibur flow cytometer was employed for detection (BD Biosciences) [19].

### 2.6. GFP-LC3 Fluorescence

Chondrocytes were plated at 2 × 10^3^/well and grown to 80–90% confluence. The number of LC3 in at least 30 cells was counted by FuGENE HD® Transfection Reagent (Promega) [20].

### 2.7. Enzyme-Linked Immunosorbent Assay (ELISA)

Chondrocyte culture supernatant was collected to measure the concentrations of tumor necrosis factor-*α* (TNF-*α*) and IL-6 by ELISA (Boster, Wuhan, China) [21].

### 2.8. RNase R and Actinomycin D Treatment

Total RNA (3 *μ*g) was incubated with 3 U/*μ*g RNase R (Epicenter, USA) and used for detection of circMELK and MELK expression.

Actinomycin-D (2 *μ*M, Gibco) was added to the cell culture medium to block transcription and total RNA was extracted for reverse transcription quantitative polymerase chain reaction analysis [22].

### 2.9. Subcellular Analysis

Nuclear and cytoplasmic fractions were isolated by PARIS kit (Life Technologies, NY, USA). circMELK, U6, and glyceraldehyde-3-phosphate dehydrogenase (GAPDH) levels in RNAs isolated from chondrocytes were quantitatively measured [23].

### 2.10. RNA Extraction and Analysis

Total RNA was extracted using the Total RNA Rapid Extraction Kit (GK3016, Generay Biotech, Hangzhou, China) and miRNA was isolated by the PureLink® miRNA Isolation Kit (K1570-01, Thermo Fisher Scientific). The concentration and purity of total RNA were detected by Thermo Nano Drop 2000, and the integrity of total RNA was detected by SDS-PAGE Agilent-2100. mRNA/circRNAs were reverse-transcribed by HiScript II Q RT SuperMix (R222-01, Vazyme, Nanjing, China) whereas miRNAs were reverse-transcribed by SuperScript™ III Reverse Transcriptase (18080085, Thermo Fisher Scientific, USA). Quantitative PCR was performed using PowerUp™ SYBR™ Green Master Mix (A25779, Applied Biosystems, USA) and CFX Connect Real-Time System (BIO-RAD, USA). The housekeeping genes were U6 and GAPDH and gene expression was calculated by the 2^−ΔΔCt^ method. All primers used are listed in Table 1 [24].

### 2.11. Western Blot

Cartilage specimens were lysed with radio-immunoprecipitation assay lysis buffer (Thermo Fisher Scientific) and collected by centrifugation. Proteins were extracted using the protein isolation kit (Invitrogen) and quantified by the bicinchoninic acid kit (Thermo Fisher Scientific). Proteins were separated by sodium dodecyl sulfate-polyacrylamide gel electrophoresis and prepared for polyvinylidene difluoride membrane- (Millipore-) covered samples which were then blocked in 5% nonfat milk. The antibodies included MYD88 (4283, Cell Signal Technology), NF-*κ*B p65 (3033, Cell Signal Technology), GAPDH (ab8245, Abcam), Lamin B (ab16048, Abcam), beclin-1 (ab62557, Abcam), and the corresponding secondary antibody. Protein bands were quantified with the ECL system (Thermo Fisher Scientific) and analyzed by Image *J* software [25].

### 2.12. RNA Pulldown

Chondrocytes were lysed in 1 ml of lysis buffer biotinylated probe and added with 3 *μ*g of Biotin-NC and Biotin-circRNA or miRNA. After 2 h, streptavidin magnetic beads (Life Technologies) were added to prepare probe-magnetic bead complexes for PCR detection [26].

### 2.13. RNA Immunoprecipitation (RIP) Assay

RIP detection was performed using the Magna RIP™ Kit (Millipore, Bedford, MA, USA) in combination with immunoglobulin G or anti-Ago2-coated beads. miR-497-5p and MYD88 levels on the beads were analyzed.

### 2.14. Luciferase Reporter Assay

circMELK and MYD88 3' untranslated region fragments, as well as their mutated fragments, were inserted into the pmirGLO vector (Promega) to establish the wild-type (wt) and mutant-type (mut) luciferase reporter vectors. miR-497-5p mimic or inhibitor, along with an established reporter vector, was cotransfected into chondrocytes with Lipofectamine 3000 (Invitrogen). Dual Luciferase Reporter Assay Kit (Promega) was employed for luciferase activity analysis [27].

### 2.15. Statistical Analysis

SPSS 21.0 (IBM, NY, USA) was used for data assessment. The normal distribution was checked by the Kolmogorov-Smirnov test, and the results were expressed as mean ± standard deviation. One-way analysis of variance was suitable for multigroup comparison, and Fisher's least significant difference *t* test was suitable for pairwise comparison. Chi-square test was feasible for the comparison of enumeration data which were reported as rate or percentage. *P* was a two-sided test, and *P* < 0.05 was considered statistically significant.

## 3. Results

We aimed to investigate the functional role of hsa_circ_0009127 (MELK) in OA and the regulatory mechanism of hsa_circ_0009127 (MELK) regulating MYD88/NF-*κ*B signaling pathway via miR-497-5p. We isolated and cultured human chondrocytes to construct an *in vitro* model and conducted a series of experiments. We found that hsa_circ_0009127 (MELK) promotes OA chondrocyte apoptosis and inhibits autophagy by regulating the MYD88/NF-*κ*B signaling axis through miR-497-5p. Therefore, our data are the first to investigate the function and mechanism of hsa_circ_0009127 (MELK) regulating the MYD88/NF-*κ*B signaling pathway through miR-497-5p in OA, providing new insights into the pathogenesis of OA.

### 3.1. circMELK Is Elevated in OA Cartilage Tissue and IL-1*β*-Stimulated Chondrocytes

**Figure 1:**
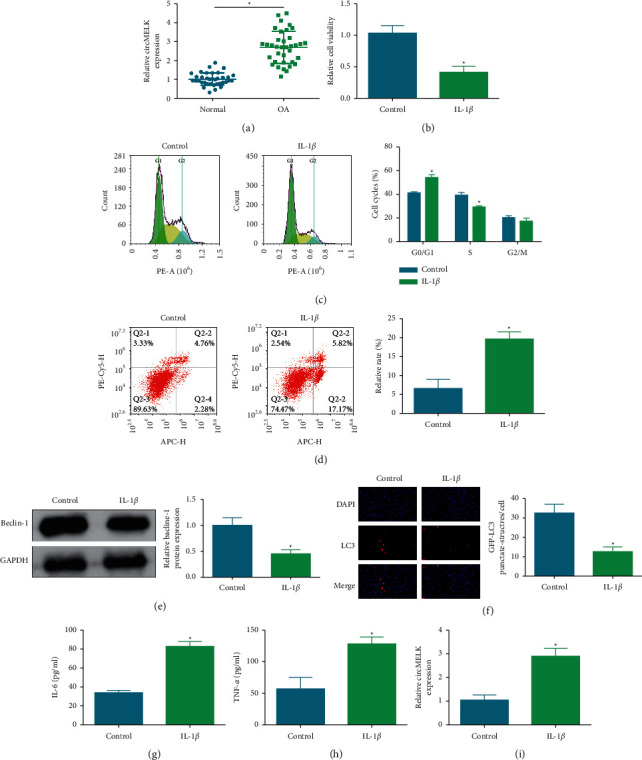
circMELK is elevated in OA cartilage tissue and IL-1*β*-stimulated chondrocytes. OA tissues expressed high circMELK (a), effects of 10 ng/ml IL-1*β* on viability (b), cell cycle and apoptosis (c)-(d), beclin-1protein expression (e), LC3 immunofluorescence (f), IL-6 and TNF-*α* concentrations (g)-(h), and circMELK expression (i) in chondrocytes. ^*∗*^*P* < 0.05.

By analyzing circMELK expression in OA cartilage specimens and normal cartilage specimens, we found that circMELK expression was elevated in cartilage specimens tissues (Figure 1(a)). A cell model established by IL-1*β* stimulation was utilized to mimic OA in chondrocytes. After IL-1*β* stimulation, it was measured that the viability of chondrocytes was impaired (Figure 1(b)), G1 phase arrest was prevented (Figure 1(c)), and apoptosis was induced (Figure 1(d)). It has been accepted that OA progression is associated with insufficient autophagy in chondrocytes [28], and the activation of autophagy is often accompanied by increased expression of LC3 and beclin-1 [29]. Western blot analysis detected that IL-1*β* decreased beclin-1 expression (Figure 1(e)), GFP-LC3 immunofluorescence presented that IL-1*β* inhibited the number of GFP-LC3 (Figure 1(f)), and ELISA results showed that IL-1*β* raised the concentrations of proinflammatory cytokines, including IL-6 and TNF-*α* in chondrocytes (Figures 1(g) and 1(h)). Taken together, the OA cell model was successfully established. Notably, it was recognized that circMELK expression was elevated in IL-1*β*-treated chondrocytes (Figure 1(i)), implicating that circMELK may act as an inducer of OA.

### 3.2. circMELK Promotes IL-1*β*-Induced Chondrocyte Apoptosis and Inhibits Autophagy

**Figure 2:**
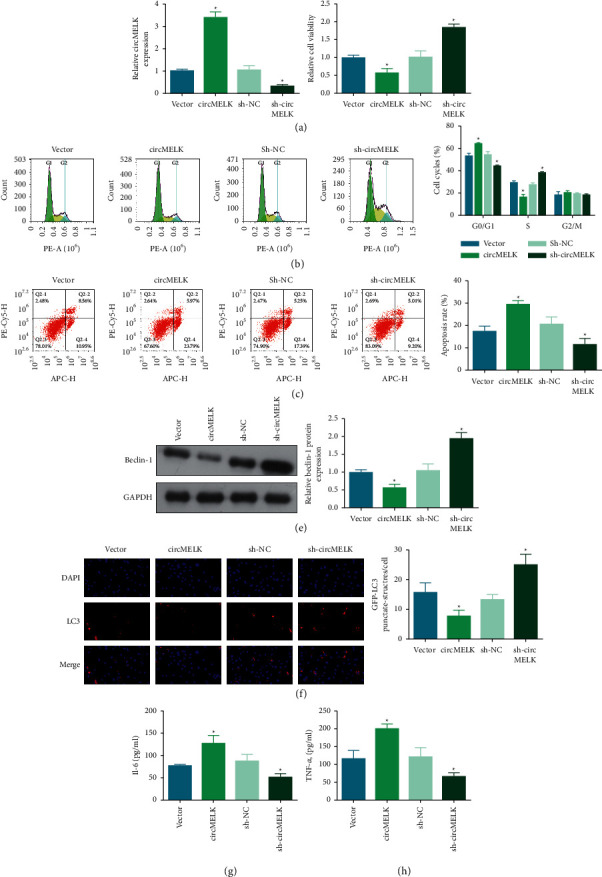
circMELK promotes IL-1*β*-induced chondrocyte apoptosis and inhibits autophagy. circMELK transfection efficiency (a), and effects of circMELK on viability (b), cell cycle and apoptosis (c)-(d), beclin-1 protein expression (e), LC3 immunofluorescence (f), and IL-6 and TNF-*α* concentrations (g)-(h) in IL-1*β*-stimulated chondrocytes. ^*∗*^*P* < 0.05.

Chondrocytes were transfected with circMELK overexpression vector or shRNA against circMELK, to overexpress or downregulate circMELK expression, respectively, and the transfection efficacy was evaluated after IL-1*β* treatment (Figure 2)(a). Subsequently, functional tests indicated that circMELK overexpression further aggravated IL-1*β*-mediated impacts on viability, apoptosis, cycle arrest, LC3 and beclin-1 expression, and concentrations of proinflammatory indices, whereas knockdown of circMELK led to the contrary results (Figures 2(b)–2)(h). Taken together, CircMELK promotes IL-1*β*-induced chondrocyte apoptosis and inhibits autophagy.

### 3.3. An Interplay between circMELK and miR-497-5p

**Figure 3:**
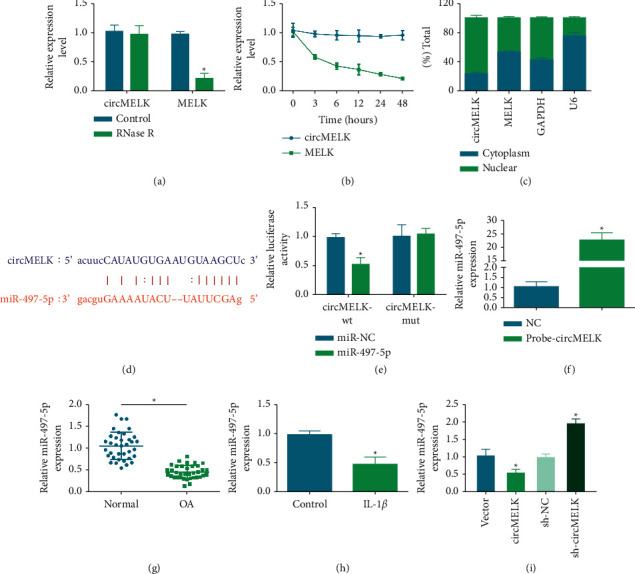
An interplay of circMELK and miR-497-5p. circMELK and MELK mRNA after RNase R treatment (a) and after actinomycin D treatment (b), and their expression in nucleus and cytoplasm (c). Binding sites of circMELK and miR-497-5p (d) and verification of the binding relation (e)-(f). miR-497-5p expression in OA patients and *in vitro* cell model (g)-(h). miR-497-5p expression after regulation of circMELK (i). ^*∗*^*P* < 0.05.

Potential miRNAs regulated by circMELK were predicted from multiple databases to elucidate the action of circMELK in OA. Firstly, the characteristics of circMELK were analyzed. After RNase R treatment, MELK mRNA levels dropped, but circMELK exhibited strong resistance to RNase R digestion (Figure 3(a)). Under the action of actinomycin D, circMELK expression was more stable than linear MELK (Figure 3(b)). Nuclear isolation experiments showed that circMELK was enriched in the cytoplasm (Figure 3(c)). miR-497-5p was identified by starbase as a downstream of circMELK for further study (Figure 3(d)). The luciferase reporter assay showed that when miR-497-5p was bound to circMELK-wt, the luciferase activity was reduced (Figure 3(e)). RNA pulldown identified the binding of circMELK to miR-497-5p, showing that miR-497-5p was pulled down by the biotinylated probe targeting circMELK (Figure 3(f)). In addition, miR-497-5p expression reduction was examined in both OA cartilage specimens and IL-1*β*-treated chondrocytes (Figures 3(g)) and 3(h)) and circMELK overexpression restricted miR-497-5p expression while circMELK downregulation induced miR-497-5p expression in IL-1*β*-treated chondrocytes (Figure 3(i)).

### 3.4. miR-497-5p Suppresses IL-1*β*-Induced Chondrocyte Apoptosis and Autophagy

**Figure 4:**
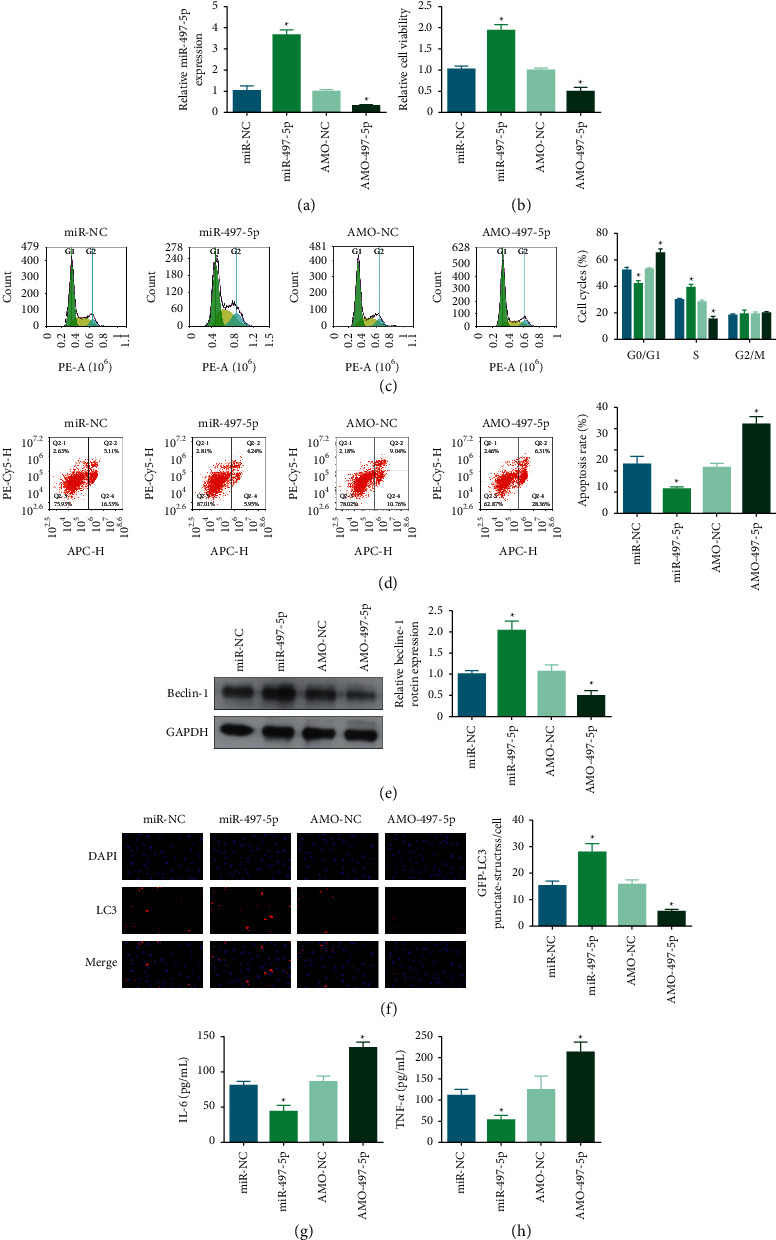
miR-497-5p suppresses IL-1*β*-induced chondrocyte apoptosis and autophagy. miR-497-5p transfection efficiency (a), and effects of miR-497-5p on viability (b), cell cycle and apoptosis (c)-(d), beclin-1 protein expression €, LC3 immunofluorescence (f), and IL-6 and TNF-*α* concentrations (g)-(h) in IL-1*β*-stimulated chondrocytes. ^*∗*^*P* < 0.05.

Pivoted on the action of miR-497-5p in OA, miR-497-5p mimic or AMO-497-5p was transfected into chondrocytes to overexpress or silence miR-497-5p expression, respectively (Figure 4(a)). The experimental results offered evidence that miR-497-5p overexpression made the reversal of IL-1*β*-induced effects (viability, apoptosis, cycle arrest, LC3 and beclin-1 expression, and concentrations of proinflammatory indices) but miR-497-5p silence further promoted IL-1*β*-induced effects on chondrocytes (Figures 4(b))–4(h)). Precisely, miR-497-5p suppresses IL-1*β*-induced chondrocyte apoptosis and autophagy.

### 3.5. A Regulatory Relation between miR-497-5p and MYD88

**Figure 5:**
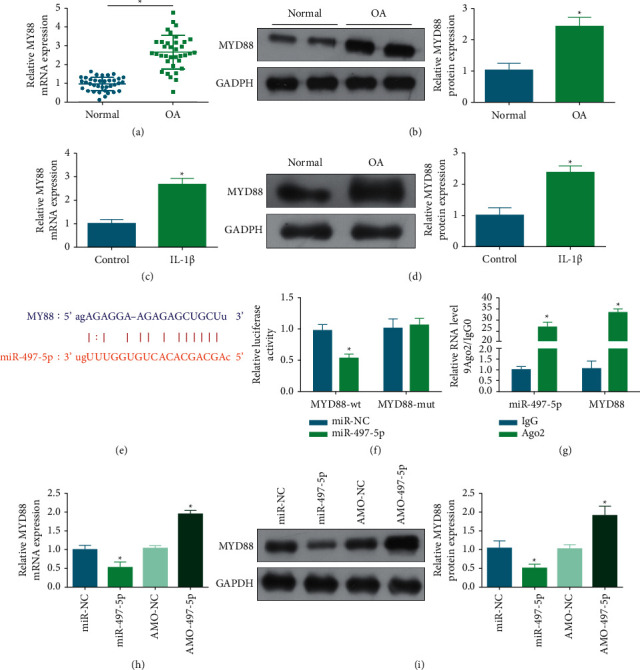
A regulatory relation between miR-497-5p and MYD88. MYD88 expression in OA tissue and cell models (a)–(d). miR-497-5p and MYD88 binding sites (e) and verification of the targeting relation (f)-(g). MYD88 expression after regulating miR-497-5p (h). ^*∗*^*P* < 0.05.

MYD88 expression was elevated in OA cartilage specimens and IL-1*β*-treated chondrocytes (Figures 5(a)–5(d)). Notably, MYD88 was first identified as a potential target of miR-497-5p by bioinformatics analysis (Figure 5(e)) and this relation was verified by detecting the luciferase activity of MYD88-wt in miR-497-5p-overexpressed chondrocytes in dual luciferase reporter assay (Figure 5(f)) and by determining the enrichment of miR-497-5p and MYD88 under Ago2 treatment in RIP assay (Figure 5)(g). Furthermore, it was noticed that miR-497-5p mimic transfection inhibited MYD88 levels while AMO-497-5p transfection induced MYD88 expression in IL-1*β*-treated chondrocytes (Figures 5(g) and 5(i)). Overall, miR-497-5p has a targeting relation with MYD88.

### 3.6. MYD88 Blocks the Effect of Overexpressing miR-497-5p on IL-1*β*-Stimulated Chondrocytes

**Figure 6:**
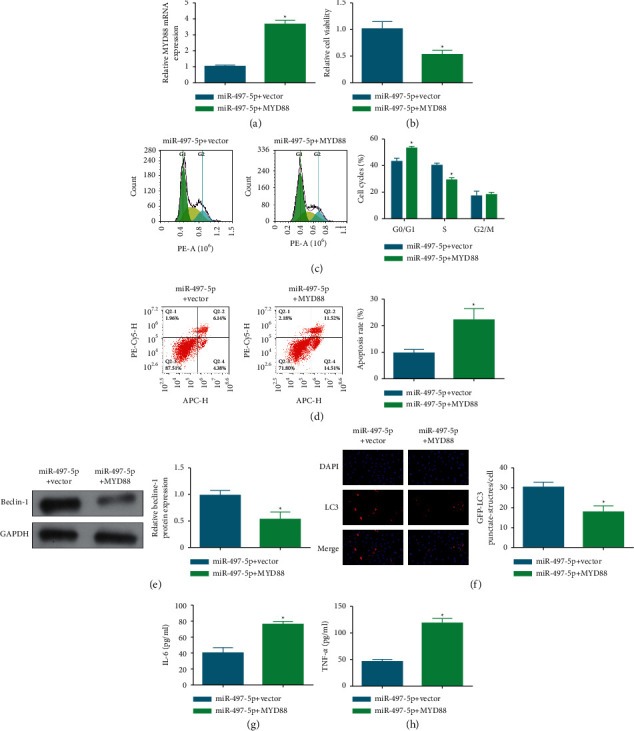
MYD88 blocks the effect of overexpressing miR-497-5p on IL-1*β*-stimulated chondrocytes. MYD88 transfection efficiency (a), and effects of MYD88 on viability (b), cell cycle and apoptosis (c)-(d), beclin-1 protein expression (e), LC3 immunofluorescence (f), and IL-6 and TNF-*α* concentrations (g)-(h) in IL-1*β*-stimulated chondrocytes. ^*∗*^*P* < 0.05.

Paying attention to the regulatory action of MYD88 and miR-497-5p in OA, we performed a rescue assay by upregulating MYD88 in IL-1*β*-stimulated chondrocytes overexpressing miR-497-5p (Figure 6(a)). Subsequent cellular experiments found that, after upregulation of MYD88, miR-497-5p overexpression-mediated chondrocyte viability (Figure 6(b)), cell cycle arrest (Figure 6(c)), apoptosis (Figure 6(d)), autophagy (Figures 6(e) and 6)(f), and inflammation (Figures 6(g) and 6(h)) were all reversed. Shortly, MYD88 blocks the effect of overexpressing miR-497-5p on IL-1*β*-stimulated chondrocytes.

### 3.7. MYD88 Activates the NF-*κ*B Pathway

**Figure 7:**
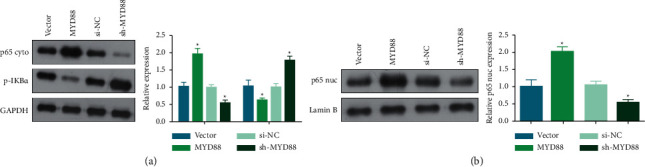
MYD88 activates the NF-*κ*B pathway. Effects of MYD88 on NF-*κ*B pathway (a)-(b). ^*∗*^*P* < 0.05.

By analyzing the activation of the NF-*κ*B pathway, we further explored the molecular mechanism of the circMELK/miR-497-5p/MYD88 axis in the progression of OA. As the data reported, MYD88 promoted the phosphorylation of IkB*α*, which facilitated the entry of p65 into the nucleus and ultimately activated the NF-*κ*B signaling pathway (Figures 7(a) and 7(b)).

## 4. Discussion

Current treatments of OA can only relieve pain and cannot prevent cartilage damage and other joint tissue destruction [30]. The secretion of inflammatory factors has been reported in the pathological process of OA; therefore, IL-1*β*-treated human chondrocytes have been used to establish inflammatory injury models to study OA *in vitro* [31]. CircRNAs have recently been confirmed to be closely related to OA disease progression [32], based on which, we studied that circMELK is a novel OA causative factor and further testified that abnormal elevation of circMELK promoted OA chondrocyte apoptosis and inhibited autophagy by regulating the MYD88/NF-*κ*B signaling axis through miR-497-5p.

Noncoding RNAs have been considered an effective way to reduce OA [33] and circRNAs function as functional RNAs, playing indispensably in OA [34]. It has been reported that articular cartilage degeneration is one of the important pathological processes of OA. Chondrocytes are the only resident cells in cartilage and maintain cartilage integrity. Chondrocyte apoptosis is an important factor in promoting OA [35]. In contrast, enhancement of chondrocyte autophagy prevents OA progression in articular cartilage [36]. Autophagy is an important protective mechanism that maintains the balance of anabolic and catabolic activities and maintains cellular homeostasis, and its dysfunction is a hallmark of OA pathogenesis [37]. Therefore, inhibiting chondrocyte apoptosis and enhancing their autophagy are a promising therapeutic strategy for OA. Our study found that circMELK silence enhanced chondrocyte viability, accelerated cell cycle, and inhibited apoptosis, whereas circMELK overexpression did the opposite, suggesting that circMELK management might be a promising strategy for OA. It is well known that circRNAs are generally involved in the regulation of many diseases including OA by acting as miRNA sponges [38, 39]. It has been documented that circMELK can act as a sponge for miRNAs, such as miR-593 [40], while in this work, miR-497-5p was an identified target of circMELK.

Aberrant expression of miR-497-5p is mainly seen in different types of cancer [41], diabetic nephropathy [42], Parkinson's disease [43], preeclampsia [44], and metabolic syndrome [45]. In particular, Zhao et al. have found that miR-497-5p can promote osteoblast differentiation in osteoporosis [46]. In addition, current reports also check the decreased levels of miR-497-5p in osteoporotic bone [47, 48] and human osteoarthritic cartilage [15]. Our study also found that miR-497-5p was downregulated in OA cartilage tissue and IL-1*β*-stimulated chondrocytes, and miR-497-5p was protective against apoptosis and autophagy of IL-1*β*-induced chondrocytes, whereas silencing miR-497-5p gave the exact opposite result.

MYD88 promotes IL-1*β*-induced inflammation in human articular chondrocytes by activating the NF-*κ*B signaling pathway [49]. NF-*κ*B signaling pathway is involved in the regulation of various biological processes and closely related to the release of inflammatory cytokines [50, 51]. Therefore, we further explored whether miR-497-5p regulates OA through the MYD88/NF-*κ*B axis. Indeed, we determined that miR-497-5p had an interplay with MYD88, detected that MYD88 was upregulated in OA chondrocytes, and confirmed that miR-497-5p inhibited MYD88 expression. Furthermore, we realized that MYD88 reversed the effect of overexpressing miR-497-5p on IL-1*β*-stimulated chondrocytes by activating the NF-*κ*B signaling pathway.

It is undeniable that there are some limitations, mainly the insufficient sample size. In addition, further *in vivo* animal experiments are required to verify the effect of the circMELK/miR-497-5p/MYD88/NF-*κ*B axis on OA progression. Finally, osteoblast dysfunction is also a key cause of OA [52], miR-497-5p has been shown to regulate osteoblast differentiation, and we hope to further explore the effect of circMELK on osteoblasts in OA in future studies to refine the molecular mechanism by which circMELK regulates OA progression.

## 5. Conclusion

All in all, circMELK regulates chondrocyte apoptosis and autophagy through the miR-497-5p-mediated MYD88/NF-*κ*B signaling pathway, thereby promoting the progression of OA. This result helps to enhance the understanding of OA pathogenesis and provides new potential targets for OA prevention and treatment [53].

## Figures and Tables

**Table 1:** Primer sequence.

Genes	Sequence (5′⟶3′)
circMELK	F: TTGAGGCCTTGAAGAACCTG
	R: CCACCTGTCCCAATAGTTTCA

miR-497-5p	F: TCGGCAGGCAGCAGCACACUG
	R: CACTCAACTGGTGTCGTGGA

MYD88	F: AAAGGCTTCTCAGCCTCCTC
	R: ACTGCTCGAGCTGCTTACCA

U6	F: CTCGCTTCGGCAGCACA
	R: AACGCTTCACGAATTTGCGT

GAPDH	F: ATGCTGCCCTTACCCCGG
	R: TTACTCCTTGGAGGCCATGTAGG

## Data Availability

The figures used to support the findings of this study are included within the article.
